# NK Cells in the Human Lungs

**DOI:** 10.3389/fimmu.2019.01263

**Published:** 2019-06-04

**Authors:** Baptiste Hervier, Jules Russick, Isabelle Cremer, Vincent Vieillard

**Affiliations:** ^1^Centre d'Immunologie et des Maladies Infectieuses, Sorbonne Universités, Université Pierre et Marie Curie Université Paris 06, INSERM U1135, CNRS ERL8255, Paris, France; ^2^Centre de Recherche des Cordeliers, INSERM UMR S1138, Université Pierre et Marie Curie, Sorbonne Universités, Université Pierre et Marie Curie Université Paris 06, Paris, France

**Keywords:** lung, NK cells, tissue-resident NK cells, CD49a, CD103

## Abstract

The lung offers one of the largest exchange surfaces of the individual with the elements of the environment. As a place of important interactions between self and non-self, the lung is richly endowed in various immune cells. As such, lung natural killer (NK) cells play major effector and immunoregulatory roles to ensure self-integrity. A better understanding of their abilities in health and diseases has been made possible over the past decade thanks to tremendous discoveries in humans and animals. By precisely distinguishing the different NK cell subsets and dissecting the ontogeny and differentiation of NK cells, both blood and tissue-resident NK populations now appear to be much more pleiotropic than previously thought. In light of these recent findings in healthy individuals, this review describes the different lung NK cell populations quantitatively, qualitatively, phenotypically, and functionally. Their identification, immunological diversity, and adaptive capacities are also addressed. For each of these elements, the impact of the mutual interactions of lung NK cells with environmental and microenvironmental factors are questioned in terms of functionality, competence, and adaptive capacities. As pulmonary diseases are major causes of morbidity and mortality worldwide, special attention is also given to the involvement of lung NK cells in various diseases, including infectious, inflammatory, autoimmune, and neoplastic lung diseases. In addition to providing a comprehensive overview of lung NK cell biology, this review also provides insight into the potential of NK cell immunotherapy and the development of targeted biologics.

## Introduction

The lung is faced daily with 10,000 liter of inhaled air containing a myriad of particles, potentially recognized as non-self. This constant exposure of one of the most important interfaces (>200 m^2^) of the body requires a fine-tuned and rapidly acting immune system to immediately sense and protect the host at this intimate contact zone. For this purpose, the airways are endowed with a broad armamentarium of cellular and humoral host defense mechanisms, most of which belong to the innate arm of the immune system. The complex interplay between resident and infiltrating immune cells acting in concert with secreted proteins, such as defensins, mucins, or collectins, shapes the outcome of host-pathogen, host-allergen, and host-particle interactions within the airway microenvironment. Among the initial checkpoints that encounter inhaled antigens and trigger pro-inflammatory or tolerogenic/anti-inflammatory downstream immune responses, natural killer (NK) cells play a key role.

As innate lymphoid cells, NK cells provide a first line defense against infection and cancer. In comparison to their classic adaptive counterparts, NK cells are considered innate short-lived effectors with a turnover time of approximately 2 weeks (1), compared to months or years for some T-cell subsets (2). Consistent with the critical nature of NK cell killing, impaired cytolysis is the primary diagnostic criterion in patients with functional NK cell deficiencies (3). NK cells also play regulatory roles via the release of cytokines and chemokines, and interactions with other immune cells. As such, they are also involved in various inflammatory and auto-immune diseases, in which they can act as protectors or promotors.

Under normal immune surveillance, NK cells express inhibitory receptors, including killer Ig-like receptors (KIRs), ILT-2, and the CD94:NKG2A heterodimer, which recognize primarily classical and non-classical major histocompatibility complex (MHC) class I molecules (4, 5). NK cell activation is possible when target cells lack expression of MHC-I molecules, a mechanism so called “missing-self” recognition. NK cell activity occurs also when stimulatory signals outweigh MHC class I inhibition. Several of these activating receptors have been characterized, including NKG2C, NKG2D, and the natural cytotoxicity receptors (NCRs) NKp30, NKp44, and NKp46, which ensure “stress-induced” recognition (4).

In addition to the description of this vast network of activating and inhibitory receptors, the knowledge of NK cell biology has improved in the past decades in terms of their maturation, diversity, and adaptive capacities (5), which are, at least in part, guided by the response to environmental factors, including non-fatal acute and chronic viral infections (6–8). More recently, following the identification of specific receptors related to tissue residency, a great step in understanding the critical role of NK cells in controlling self and non-self has been taken. Indeed, more than NK cells from peripheral blood, NK cells from tissues are directly interacting with normal and abnormal (micro)environments. As such, the lung contains a high reservoir of NK cells. Although still poorly understood, studies of NK cells within this organ, both in normal and pathological situations in humans, would tremendously increase the knowledge of NK cell biology. According to these recent advances, the development of new therapeutic targets could emerge, leading to a better management of respiratory diseases, which are one of the leading causes of death worldwide.

To this end, this mini-review will focus only on certain areas, with the aim of describing the specific roles of NK cells in the lung based on the most recent and exciting advances in health and disease.

## NK cells in the Normal Lung

### Identification of NK Cell Populations

**Figure 1:**
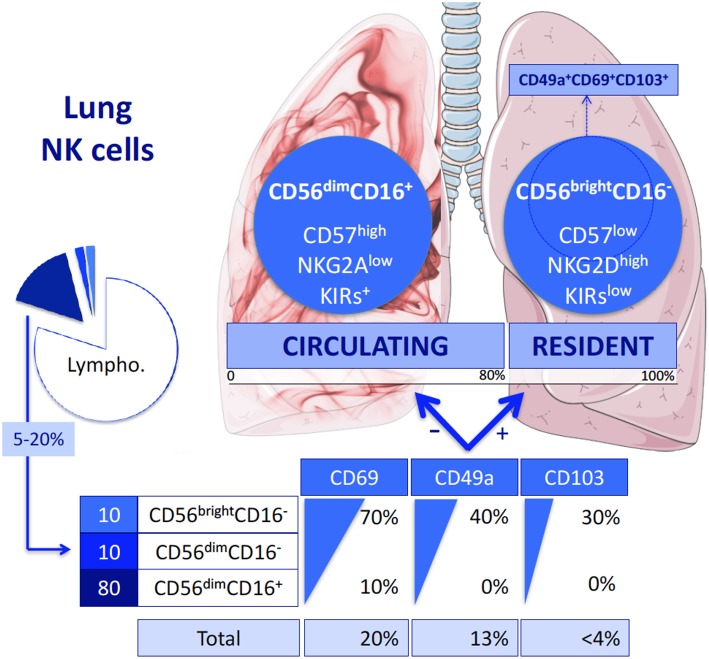
Lung NK cell subpopulations. Like peripheral blood NK cells, lung NK cells represent 20% of all the Lymphocytes and are composed of three different subsets: CD56^dim^CD16^+^, CD56^dim^CD16^−^, and CD56^bright^CD16^−^ NK cells. Each subset expresses three markers of residency differentially. As a result, most of the lung NK cells do not express these markers: they form the circulating NK cells. They belong to the CD56^dim^CD16^+^ population and disclosed a terminally differentiated phenotype. In contrast, the cells expressing CD69, CD49a, and/or CD103 are considered as being resident NK cells. Almost all of them are CD56^bright^CD16^−^ or in a lesser extend CD56^dim^CD16^−^ NK cells. They display a less mature phenotype. Among them, triple positive CD49a^+^CD69^+^CD103^+^ are thought to be more specifically the resident population, representing *in fine* < 3% of the total lung NK cells.

Despite a princeps study of NK cells in the human lungs in the 1980s (9), these cells have only recently been characterized in normal lungs (10, 11). The proportion of NK cells in this organ is roughly similar or even slightly higher than in peripheral blood, ranging from 5 to 20% of the CD45^+^ lymphocytes (10). As shown in Figure 1, the vast majority (up to 80%) of lung NK cells display a mature CD56^dim^CD16^+^ phenotype (10, 11). The remaining subsets are composed of immature CD56^bright^CD16^−^ and CD56^dim^CD16^−^ cells, this latter corresponding either to an intermediate stage of differentiation (12) or to recently activated NK cells that have lost cell-surface CD16 expression (13). These data contrast with NK cells from other tissues, including liver and secondary lymphoid organs, in which the CD56^bright^CD16^−^ subset largely predominates (14–16). Thus, in the lung, the different populations are present in similar proportions than in the peripheral blood, suggesting that most NK cells in the lungs are circulating cells. As a whole, this raises the question of the existence of resident lung NK cells *vs*. circulating cells, and of their identification.

**Figure 2:**
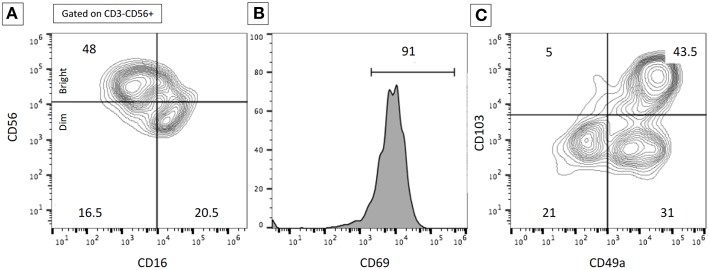
Example of flow cytometry data illustrating the subset of resident lung NK cells. Flow cytometry analyses were performed on BALF in a patient with severe interstitial lung disease. The expression of the cell surface markers was performed after gating on CD3^−^CD56^+^ NK cells. **(A)** Proportions of CD56^dim/bright^ and CD16^+/−^ NK cells. **(B)** High expression of CD69^+^ on NK cells. **(C)** Proportions of resident NK cells according to CD103 and CD49a expression. The proportion of resident lung NK cells was higher than expected on normal lung samples. Numbers represent the % of the different populations.

By analogy with tissue resident T lymphocytes, resident lung NK cells were first identified by the cell surface expression of CD69 (17, 18), which is involved in maintaining immune cells within organs through inhibition of sphingosine-1-phosphate receptor. CD69^+^ was differentially expressed in lung and matched peripheral blood NK cells (10). The subset of CD69^+^ NK cells represents ~25% of the total of lung NK cells. More recently, and in light of data regarding NK cells as well as T cells within other tissues (17), a more precise characterization of resident lung NK cells has been proposed. This identification is based on CD49a, known as a1-integrin (11, 19), which is not expressed by NK cells in the peripheral blood. Based on this definition, tissue resident lung NK cells reach up to 15% of lung NK cells. In their study, Cooper et al. (11) also analyzed the expression of CD69 and of a third marker of residency among NK cells, the aE-integrin also known as CD103. Both markers are differentially expressed by blood and lung NK cells. Not surprisingly, the CD49a^+^ resident NK cells significantly express both CD69 and CD103 in much higher proportions than CD49a^−^ NK cells. Of note, these different markers of lung residency are mostly expressed by the immature CD56^bright^CD16^−^ and CD56^dim^CD16^−^ NK cell subsets, whereas they are only slightly expressed by mature CD56^dim^CD16^+^ NK cells. Based on this observation, it has been suggested that the small subset of triple positive CD49a^+^CD69^+^CD103^+^ NK cells (Figure 2) could define resident NK cells more specifically (11).

From these definitions, it could be considered as a whole that resident NK cells represent the minority of lung NK cells (one-quarter of lung NK cells at most). Notably, this fraction in the lung is significantly smaller than that of other tissues, such as the liver in which resident NK cells represent 50% of their total (16). These data also indicate that the vast majority of lung NK cells (the remaining three-quarters) are circulating NK cells, which are mainly CD56^dim^CD16^+^ NK cells (10).

### Phenotypical and Functional Characterization of Lung NK Cells

In-depth phenotypical analyses of lung NK cells have been performed among the different lung NK cell subpopulations to assess their maturation profile. This has been done according to previous studies showing that educated NK cells expressing KIRs and CD57 in association with low expression of NKG2A (12) would characterize the mature peripheral blood NK cell subset. It is difficult to perform such studies among each subpopulation (with respect to their resident or circulating characteristics) because dissecting them generates small groups, although this may be possible in the future with the use of mass cytometry. To date, flow cytometry analyses have focused on lung CD56^dim^ NK cells, considered herein as being mainly circulating lung NK cells, revealing that they disclose a terminally differentiated phenotype (10). It has not yet been clearly determined yet whether the proportion of these matured NK cells is enriched within the lungs or as frequent as observations made in the periphery (10, 11). In addition, these NK cells are hypofunctional against target cells when considering natural cytotoxicity, ADCC, and GM-CSF, spontaneously or following stimulation with PMA and Ionomycin or IFNα treatment (10, 11).

By contrast, when focusing on resident lung CD69^+^CD56^dim^ or CD56^bright^ NK cells, it appears that these subpopulations have not matured to a terminal stage and are phenotypically similar to their blood counterpart (10). Interestingly, the CD49a^+^CD56^bright^ resident NK cells show a higher capacity to degranulate and to produce interferon (IFN)-γ when in contact with virally infected autologous macrophages *in vitro*, as compared to matched peripheral NK cells (11).

As a whole in the lung, the predominant circulating NK cells are highly differentiated but hypofunctional, while resident lung NK cells have the capacity to be hyperfunctional.

### Diversity, Education, and Memory of Lung NK Cells

With recent technological advances, such as mass cytometry and single cell RNA sequencing (20), NK cell diversity has been extensively described and now appears dramatically much more important than previously expected; based on 28 surface markers, the NK cell repertoire is composed of up to 3 × 10^4^ subpopulations (21). Globally, this diversity should be more important if the NK cell repertoire is settled at the different levels (22), including NK cell development, differentiation, and maturation (12, 21), and also the different functional capacities, to finally promote efficient innate immune response against a large variety of stress situations. While NK cell diversity is partly determined genetically (combination, number, and polymorphisms of KIRs), its modulation throughout life is mediated by interactions with the tissue microenvironment (23). As such, the lung offers one of largest interfaces with elements of the outside environment and with the microbiota. Although little is known to date regarding lung microbiota in humans, its impact on adaptive immunity and lung diseases have been suggested (24, 25). Despite correlations between microbiota and cytokines at least produced by NK cells, such as TNFα, a direct effect on lung NK cell activation or diversity has not yet been demonstrated (26). Furthermore, lung tissue consists of many different immune and non-immune cell types, thus offering many possibilities for acquiring NK cell diversity, both in normal and pathologic situations. Unlike other tissues, the lung NK cell diversity and its acquisition have been very little studied, especially regarding the resident lung populations. NK cell diversity is, however, perceptible even for the main resident population within the lung, namely CD49a^+^CD56^bright^CD16^−^ NK cells. According to the residency markers CD69 and CD103, four different resident subpopulations may be distinguished. The CD69^+^CD103^+^ subset is the most important as compared to single positive or double negative subsets (11). The respective significance of these subsets in terms of ontogeny, differentiation, or functionality remains to be deeply studied.

Effector functions of NK cells are mainly governed by receptor interactions with MHC molecules. This process, so called “education,” is essential for ensuring diversity and local immune surveillance in the lung against different stress situations, including cancer development (27). Phenotypical analyses of KIR expression by both circulating and resident NK cells in the normal lung have clearly demonstrated the presence of “educated” cells (10). These data suggest that the observed hypo-functionality of the circulating subset seems not to be related to a default in the process of education.

The identification of adaptive subpopulations among resident lung NK cells remains unknown, but could provide essential informations to search for the constitution of a memory NK cell signature in the lung. As previously described, most of the memory NK cells were derived from the expansion of adaptive CD57^+^NKG2C^+^ cells in a context of cytomegalovirus seropositivity (6, 7). Although NKG2C overexpression has not been demonstrated in lung NK cells from healthy donors (11), it could be hypothesized that, as being the site of many viral infections, the lung would be an interesting tissue in which to study the acquisition of NK cell memory (28).

## Lung NK Cells in Diseases

Deciphering the distinct roles of lung NK cells in different pathological situations would help in understanding their complex functionality. However, studies distinguishing the roles played by resident vs. circulating lung NK cells in lung diseases, which requires matched and complex samples (including peripheral blood, BALF, and/or lung biopsy), have not yet been performed.

### Quantitative Modulation of Lung NK Cells

Regarding the number of NK cells in the lung, one consideration that might be taken thus far is that the proportions of lung NK cells and their subpopulations do not appear to vary throughout life (11). By contrast, witnessing the possible impact of (micro)environmental factors, active cigarette smoking, and to a lesser extent, past smoking habit, decrease the number of lung NK cells (10, 11), whereas they are rapidly and dramatically increased during influenza virus lung infection. In different inflammatory diseases, including sarcoidosis, COPD, hypersensitivity pneumonitis (29), autoimmune diseases, and idiopathic pulmonary fibrosis, however, conflicting results have been found regarding the proportions and number of NK cells within the lungs (Table 1). Irrespective of their circulating or resident nature, NK cells might be increased or decreased during these diseases. These differences could have many causes, which may affect trafficking, homing, or local proliferation (10). Some have been slightly studied in mice (40, 41), but have not yet been explored in detail in humans.

**Table 1:** Specificities of lung NK cells in non-neoplastic respiratory diseases.

**Diseases**		**Quantitative observations**	**Qualitative considerations**	**References**
Systemic Auto-immune Diseases	Sjögren Syndrome	Normal absolute count of Activated (HLA-DR^+^) NK cells in BALF	nd	(30)
	Systemic Sclerosis	Normal absolute count of Activated (HLA-DR^+^) NK cells in BALF	nd	(30)
	Anti-synthetase Syndrome	NK cells infiltration of all areas of lung fibrosis	NK cell expression of Granzyme B, Significant CD69 expression	(31, 32)
Inflammatory Diseases and/or Fibrosing diseases	Behçet Disease	Lower proportion of NK cells in BALF	Lower cytotoxicity	(33)
	Sarcoidosis	Increased number of CD56^bright^ NK cells in BALF	Immature phenotype with NKG2A^high^ & KIR^low^ predominant phenotype. Higher capacity to produce IFN-γ and TNF-α cytokines	(34–36)
	COPD	Normal count of CD56^+^CD16^+^ lung NK cells	Decreased CD8 expression associated with poor outcome, Higher cytotoxicity. Normal expression of the activating receptor NKG2D but abnormal expression of its ligands MICA/B by lung epithelial cells.	(37)
	Idiopathic Pulmonary Fibrosis	Presence of NK cells in BALF	Predisposing factor involving NKG2D-MICA/B pathway	(38)
Allergy	Asthma Hypersensitivity Pneumonitis	Decreased proportion of CD56^dim^ NK cells in BALF Higher number of NK cells in BALF	Increased expression of Granzyme A nd	(39) (29)
Infectious Diseases^*^	Influenza A Virus^**^	nd	Resident NK cells are hyperfunctional after *ex vivo* Infection, including degranulation, granzyme B expression and IFN-gamma expression	(11)
	HCMV^°^	Higher proportion of lung NKG2C+ NK cells (BALF) of patients with HCMV viremia following lung transplantation	NKG2C+ NK cells are more mature and have higher proliferation capacities. All abnormalities are associated with poor outcomes.	(28)

HLA, Human Leukocyte Antigen; BALF, Broncho-alveolar lavage fluid; nd, not determined; HCMV, Human Cyto-megalo-virus; COPD, chronic obstructive pulmonary disease;

* focuses on human studies although animal models exists for various infections (including Mycobacterium Tuberculosis, Klebsiella Pneumoniae…).

** first infectious disease in which analyses have been performed according to the definition of resident lung NK cells,

° *studies are available only in the context of lung transplantation*.

### Phenotypical and Functional Changes of Lung NK Cells in Inflammatory Diseases

In addition to their number, the phenotype and function of NK cells could also provide information regarding their involvement in diseases. As natural cytotoxicity has been shown to be influenced by cigarette smoke, it has been also hypothesized that the functionality of lung NK cells (10) could be influenced by environmental factors as well as by the lung microenvironment. Indeed, broncho-alveolar epithelial cells produce interleukin-15 during inflammation (42), whereas alveolar macrophages, the main population of immune cells within the lungs, are known to produce soluble factors likely to alter NK cell functions, such as transforming growth factor-β (43), following environmental toxin exposure.

Sarcoidosis (34–36) is a systemic granulomatosis of unknown origin commonly involving the lungs. During sarcoidosis, the analyses of lung NK cells from BALF showed an increased number of CD56^bright^ NK cells, disclosing an immature phenotype of NKG2A^++^KIR^low^ NK cells. Following unspecific stimulation, these lung NK cells produce a large amount of Th1 cytokines (IFN-γ and TNF-α). Whether this population belongs to resident or circulating NK cells has not yet been determined. The consequences of these observed variations have not been explored either, especially in terms of fibrosis promotion (25).

COPD is closely associated with cigarette smoking, and is associated with recurrent infections, destruction of the lung parenchyma (emphysema), and/or airway obstruction. Both quantitative and qualitative lung NK cell abnormalities have been described in patients with COPD (37), but they have not been analyzed with respect to the recent resident or circulating definition. Despite effects opposite to those attributed to smoking, lung NK cell cytotoxicity could be enhanced in patients with COPD, especially against epithelial cells expressing the NKG2D stress ligands MICA/B. An association between enhanced stress-induced cytotoxicity and COPD severity has been observed, supporting a deleterious effect of lung NK cells in injuring self and promoting emphysema.

Deleterious involvement of stress-induced recognition could also play a role in the pathogenesis of pulmonary fibrosis: a possible predisposing factor involving the NKG2D/MICA-B pathway has been identified in patients with idiopathic pulmonary fibrosis (38). Similarly, during anti-synthetase syndrome, an autoimmune connective tissue disease associated with interstitial lung disease, the NKp30 (NCR-3)/BAT-3 axis could promote the disease (31).

Further studies are required to precisely determine the respective roles of the different lung NK cell subsets (resident vs. circulating ones) in these phenomena. Increasing our understanding of the interactions between lung NK cells and the (micro)environment (44), as well as the role of resident vs. circulating lung NK cells in maintaining immune tolerance, could also lead to therapeutic strategies targeting NK cells in these pathological situations.

### Lung NK Cells in Infectious Diseases and Cancer

Immune diversity, and NK cell diversity in particular, are essential to ensure effective recognition of the non-self. As an interface with the environment, the lung is the location of numerous infectious diseases, related to all types of pathogens. The impact of successive lung infections affecting individuals throughout life in terms of resident NK cell diversity and memory acquisition is one of the most challenging subjects of study to date. Unfortunately, no study of this kind is yet available in humans.

NK cell response to influenza virus has, however, been largely studied in mice, in which protective or detrimental effects were successively reported due to differences in influenza strain, dose, and genetic background of the mice. In humans, the majority of studies investigating NK cell response have used peripheral blood NK cells from patients or healthy donors following an *in vitro* infection (10). Notably, the specific response of resident lung CD56^bright^CD49a^+^ NK cells to influenza virus infection has been recently explored *in vitro* (11). In response to influenza infection, resident NK cells provided significant antiviral activity following contact with influenza-infected cells, natural cytotoxicity, and IFN-γ release. These data suggest that NK cell memory of influenza infection could exist within the human lung. The role of viral proteins, especially those which are bound by NKp46 and NKp44 (45), such as hemagglutinin, remains to be studied in light of the resident lung NK cell definition. Deciphering the mechanisms governing lung NK cell activation in this context, including cytokine signatures, activation pathways or transcription factors, would be of interest. In addition, both the diversity of the resident lung NK cell repertoire and the adaptive capacities of this specific lung NK cell population remain to be investigated.

Several lines of evidence also support the notion that NK cells play an important role in the control of tumor growth. Early studies dedicated to NK cell infiltration of the tumor microenvironment (TME) of non-small-cell lung carcinoma (NSCLC) suggested that NK cell density correlated with overall survival (46, 47). However, the most recent studies using the marker NKp46 (rather than the non-specific marker CD57) or the specific gene expression signature did not show any clear association between local NK cell infiltration and the clinical outcome (48–50). This could be explained by the ability of the TME to locally alter the intra-tumoral NK cell phenotype, as has been shown in different studies comparing them to matched normal lung NK cells and/or to peripheral blood NK cells. In humans, the NK cell population observed in NSCLC displayed profound alterations in the expression of relevant NK cell receptors, and more specifically, downregulation of expression of NKp30, NKp80, DNAM1, and CD16, as well as upregulation of NKG2A when compared to the normal counterpart (48, 51). Functionally, intra-tumoral NK cells displayed impaired ability to degranulate and to produce IFN-γ (48). The influence of the TME has been further confirmed by microarray analyses showing a modulation of the transcriptional profile and revealing a specific signature for intra-tumoral NK cells (52). Nevertheless, these conclusions were mainly drawn by considering NSCLC-infiltrating NK cells as a whole population. Although NK cells from the TME largely express CD69 (48), previously defined as a marker of residency, none of these studies suggested the possibility of specific modulation of tissue-resident NK cells. According to CD49a expression, such a comparison could now be more easily performed among the NK cell tumor infiltrate.

Apart from these lung residency considerations, it is also important to note that modulations of educated KIRs by intra-tumoral NK cells is a key element of tumor immune surveillance. Interestingly, the exposure of NK cells to exogenous MHC-I in mice led to upregulation of the activating receptors NKp46 and NKG2D and to downregulation of Ly49C/I inhibitors (the murine equivalent of KIRs inhibitors in humans) leading to a control of tumor growth (53). Thus, in addition to the recent development of anti-tumoral immunotherapies, which only partially affect NK cells, reversing the immunosuppressive TME to restore NK cell activity would increase the number of therapeutic strategies. Furthermore, diverse novel approaches, such as adoptive transfer of autologous, allogeneic, or engineered NK cells are also currently in development (54).

## Conclusions and Perspectives

In recent years, progress has been made in the characterization of NK cells in the lung; however, the concept of tissue resident NK cells has only recently been widely accepted, especially with the identification of residency markers, such as CD49a. These cells show important differences with the circulating NK cells in terms of phenotype and functions, which likely reflect the impact of the local micro-environment in shaping the tissue-specific characteristics of resident NK cells. The question of the ontogeny of tissue-resident NK cells remains complex and only partially explained (14), especially in the lung. While it is agreed that CD34^+^ NK cell progenitors reside in the bone marrow, there is a less clear understanding of the mechanisms controlling seeding of NK cells within the tissues. Whether seeding of these cells into organs generates tissue-specific NK cell maturation, or whether predefined common lymphoid progenitors with specific developmental and homing characteristics (55, 56) would exit under certain conditions from the bone marrow and specifically seed into the secondary lymphoid organs and finally into final sites of maturation remains unknown. Further analyses of the lung following human allogenic lung transplantation and/or graft vs. host disease in the lung following bone marrow transplantation would help improve our understanding of lung NK cell ontogeny. Armed with this knowledge, NK cell-based therapeutics (57–59) could be a promising avenue for the treatment of cancer and self/non-self-inflammation.

## Author Contributions

All authors were involved in reading bibliography and writing the article. All co-authors reviewed the article. BH drew the Figure 1 and Figure 2 which are original.

### Conflict of Interest Statement

The authors declare that the research was conducted in the absence of any commercial or financial relationships that could be construed as a potential conflict of interest. The reviewer FV and handling editor declared their shared affiliation at the time of review.

## Abbreviations

KIR: Killer Immunoglobulin-like Receptor
MHC-I: Major Histocompatibility Complex- I
GM-CSF: Granulocyte-Macrophage Colony-Stimulating Factor
NSCLC: Non-Squamous-Cell Lung Carcinoma
BALF: Broncho-Alveolar-Lavage Fluid
TME: Tumor Micro-Environment
ADCC: Antibody-Dependent Cellular Cytotoxicity
COPD: chronic obstructive pulmonary disease
TNF: Tumor-necrosis Factor
IFN: Interferon
PMA: Phorbol 12-Myristate 13-Acetate.
